# Use of the Protein Ontology for Multi-Faceted Analysis of Biological Processes: A Case Study of the Spindle Checkpoint

**DOI:** 10.3389/fgene.2013.00062

**Published:** 2013-04-26

**Authors:** Karen E. Ross, Cecilia N. Arighi, Jia Ren, Darren A. Natale, Hongzhan Huang, Cathy H. Wu

**Affiliations:** ^1^Center for Bioinformatics and Computational Biology, University of DelawareNewark, DE, USA; ^2^Protein Informatics Resource, Georgetown University Medical CenterWashington, DC, USA

**Keywords:** protein ontology, biocuration, phosphorylation, spindle checkpoint, kinetochore

## Abstract

As a member of the Open Biomedical Ontologies (OBO) foundry, the Protein Ontology (PRO) provides an ontological representation of protein forms and complexes and their relationships. Annotations in PRO can be assigned to individual protein forms and complexes, each distinguishable down to the level of post-translational modification, thereby allowing for a more precise depiction of protein function than is possible with annotations to the gene as a whole. Moreover, PRO is fully interoperable with other OBO ontologies and integrates knowledge from other protein-centric resources such as UniProt and Reactome. Here we demonstrate the value of the PRO framework in the investigation of the spindle checkpoint, a highly conserved biological process that relies extensively on protein modification and protein complex formation. The spindle checkpoint maintains genomic integrity by monitoring the attachment of chromosomes to spindle microtubules and delaying cell cycle progression until the spindle is fully assembled. Using PRO in conjunction with other bioinformatics tools, we explored the cross-species conservation of spindle checkpoint proteins, including phosphorylated forms and complexes; studied the impact of phosphorylation on spindle checkpoint function; and examined the interactions of spindle checkpoint proteins with the kinetochore, the site of checkpoint activation. Our approach can be generalized to any biological process of interest.

## Introduction

Understanding the meaning of data is essential for accurate scientific analysis and interpretation. Ontologies formalize the meaning of terms using a defined vocabulary that facilitates the integration of data and knowledge (Gkoutos et al., 2012). Interoperability of ontological resources is required to automatically analyze data across different data repositories and to enable automatic reasoning for knowledge discovery (Hoehndorf et al., 2011). The Open Biological and Biomedical Ontologies (OBO) Foundry is a collaborative initiative^1^ whose goal is to create and maintain an evolving collection of non-overlapping interoperable ontologies that will offer unambiguous representations of the types of entities in biological and biomedical reality (Ceusters and Smith, 2010). The OBO Foundry establishes best ontology practices, including adoption of a common formal language, high standards for documentation, and collaborative development (Smith et al., 2007).

Within the Foundry, the Protein Ontology (PRO^2^) is charged with the formal representation of protein-related classes (Natale et al., 2011). PRO has three sub-ontologies informally referred to as ProEvo, ProForm, and ProComp. Classes in ProEvo represent proteins that are evolutionarily related based on full-length sequence similarity. Classes in ProForm include species-specific and species-independent classes of protein isoforms, co- and post-translationally modified (PTM) forms, and variant forms. Finally, classes in ProComp encompass protein-containing complexes with formal descriptions of their components, facilitating robust annotation of variations in composition and function contexts for protein complexes within and between species (Bult et al., 2011).

Protein Ontology terms are labeled with categories to reflect their position in the PRO hierarchy. These categories are: (i) family: protein products of a distinct gene family arising from a common ancestor; (ii) gene: the protein products of a distinct gene; (iii) sequence: protein products that have a distinct sequence upon initial translation; and (iv) modification: protein products derived from a single mRNA species that differ because of some change (or lack thereof) that occurs after the initiation of translation (co- or post-translational; Natale et al., 2011).

To facilitate reliable communication and management of data, PRO is organized under the umbrella of the Basic Formal Ontology (BFO), a top-level formal foundational ontology in the biomedical domain. BFO represents, in consistent fashion, the upper level categories common to ontologies developed in different domains and at different levels of granularity. It adopts a view of reality as comprising (1) continuants: entities that continue or persist through time (objects, qualities, and functions), and (2) occurrents: the events or happenings in which continuants participate^3^. In this schema, PRO falls under continuants (object) at the molecule level. The relations used in PRO are defined in the OBO Relation Ontology (Smith et al., 2005), an ontology commonly used among the OBO Foundry ontologies.

Moreover, PRO interoperates seamlessly with other OBO ontologies by reusing terms whenever the classes needed already exist in other ontologies. This is the case for the protein complex terms found in the Cellular Component branch of the Gene Ontology (GO; Ashburner et al., 2000), which provides the species-independent protein complex terms for PRO. Therefore, most of the terms in ProComp are children of GO terms. Similarly, other ontologies are used for the logical definition of PRO terms. In particular, the Protein Modification Ontology (PSI-MOD; Montecchi-Palazzi et al., 2008) is used for amino acid residue modification terms, and NCBI taxonomy^4^ is used for species terms.

In addition, PRO leverages and cross references data in existing protein-centric informatics resources. For example, UniProtKB (Bult et al., 2011) is the main source for species-specific protein and isoform terms, and Reactome (Croft et al., 2011) is the main source for human protein complexes and protein modified forms. In this way, PRO offers the ontological representation for the entries in these resources, facilitating data integration.

The formal definition of protein forms and complexes at various levels of granularity in the PRO framework provides a means to associate annotations to the most appropriate class, as opposed to the traditional gene-level-only association. This is especially useful, for example, in cases where functions are realized by protein complexes rather than their individual components, or by specific isoforms of a protein, or by a protein modified form. Class-specific annotations are stored in PRO using controlled vocabularies and are integrated in the PRO website so they can be searched. Therefore, the PRO framework, along with the annotation and the mapping to relevant bioinformatics resources help to answer biologically important questions, such as: (1) What proteins and complexes are involved in a particular process? (2) What proteins and complexes are conserved in a given set of species? and (3) What function(s) is associated with a given protein form or complex?

To be able to answer the questions described in the previous section, PRO has to provide an adequate coverage of terms and annotations that pertain to the biological questions being asked. The ultimate goal in PRO is the representation of protein-related terms for the 12 GO Reference Genomes and human protein complexes from Reactome. Release 32.0 contains 35,196 PRO terms from which about 25,000 are ProEvo terms (family and gene-level classes), 9,500 are ProForm terms (isoforms and modified forms), and 393 are ProComp terms. In terms of annotations, there are 2,941 GO annotations derived from 1,242 publications. The distribution files^5^ include the ontology in OBO format (pro.obo), the accompanying annotation file (PAF.txt) in a tab-delimited format, and mappings to external databases, also tab delimited. PRO is also available in OWL format through BioPortal at the National Center for Biomedical Ontologies (NCBO; Musen et al., 2012).

In this article we use the features of PRO, including a graphical representation of the PRO hierarchy, to explore the spindle checkpoint. The spindle checkpoint monitors interactions between kinetochores and spindle microtubules during mitosis and meiosis and inhibits the onset of anaphase until all kinetochores have made correct attachments to the spindle (Zich and Hardwick, 2010; Sun and Kim, 2012). A functional spindle checkpoint is necessary for high fidelity chromosome segregation; loss of the checkpoint increases the incidence of aneuploidy, a condition associated with cancer and birth defects in humans. The spindle checkpoint is well conserved in eukaryotes and depends on seven core checkpoint proteins called BUB1, BUB1B (BubR1), AURKB (Aurora B), TTK (Mps1), MAD1L1, MAD2L1, and BUB3 in humans (Oh et al., 2010; Zich and Hardwick, 2010). The target of the checkpoint is the Anaphase-Promoting Complex/Cyclosome (APC/C), a multi-subunit ubiquitin ligase whose activity is required for the metaphase to anaphase transition. In the presence of an incomplete or defective spindle, the MCC, a protein complex consisting of the checkpoint proteins BUB1B, BUB3, and MAD2L1 and the APC/C component Cdc20 associates with the APC/C and inhibits its activity (Lara-Gonzalez et al., 2012).

The spindle checkpoint represents a rich use case with features to demonstrate the application of all three sub-ontologies of PRO. First, it has been extensively studied in a range of organisms, and the core checkpoint proteins are conserved in eukaryotes from yeast to humans. Thus, using ProEvo as a guide to the evolutionary relationships amongst spindle checkpoint proteins, it is possible to make predictions about checkpoint proteins based on evidence concerning their counterparts in other organisms. The ProEvo representation can also highlight differences between spindle checkpoint proteins that may have implications for checkpoint function. Second, the spindle checkpoint is highly dependent on phosphorylation – of the seven core spindle checkpoint proteins in vertebrates, three (BUB1, AURKB, and TTK) are confirmed protein kinases and all seven are phosphoproteins (Oh et al., 2010; Zich and Hardwick, 2010). The individual representation and annotation of modified protein forms in PRO facilitates studies of the role of phosphorylation in the checkpoint. Finally, spindle checkpoint proteins participate in numerous protein complexes, which can be captured by ProComp. Through our analysis we demonstrate that PRO can provide a logical framework to represent existing knowledge about proteins and complexes involved in a biological process and serve as a platform for making predictions for further experimental studies.

## Methods

### Population of PRO with spindle checkpoint information

#### Literature and data mining

Information about spindle checkpoint protein forms and their functions was identified through curation of full-length articles that were returned in a PubMed search using the keywords “Bub1,” “BubR1,” and “Mad3” (BubR1 is a commonly used synonym for the checkpoint protein BUB1B and MAD3 is the closest yeast relative of BUB1B). Because of our interest in phosphorylation of checkpoint proteins, we focused our curation efforts on the subset of articles that were flagged by the text mining tool Rule-based LIterature Mining System for Protein Phosphorylation (RLIMS-P) as containing mentions of phosphorylation in the abstract (Yuan et al., 2006). We extracted information on all proteins for which there was experimental data in the articles we curated, thereby expanding our analysis of the checkpoint beyond the three proteins we used as keywords for the PubMed search. In addition, we mined three curated interaction databases [Molecular INTeraction Database (MINT^6^; Chatr-Aryamontri et al., 2007; release date 10/26/2012); IntAct^7^ (Kerrien et al., 2012; release 159); and the Biological General Repository for Interaction Datasets (BioGRID^8^; Stark et al., 2011; release 3.1.94)] for all direct physical interactions that had been demonstrated in low throughput experiments involving proteins identified in our literature search.

#### RACE-PRO: PRO community annotation interface

All information on protein forms was entered into Rapid Annotation interfaCE for PRO (RACE-PRO^9^), a web-based interface for PRO community annotation. This interface is intended for any user independent of their ontology knowledge. It allows the specification of a protein form by entering the protein sequence and features (protein regions, and/or modified residues) with the evidence source (usually literature), and the functional annotation associated with the given protein form using controlled vocabularies, such as GO for processes, functions, and subcellular location, and Pfam^10^ (Punta et al., 2012) for protein domains. Currently, RACE-PRO cannot be used for protein complex or protein family terms, although an expanded version of RACE-PRO that would enable these capabilities is under development. Instead, a user can request complex and family terms via the SourceForge PRO tracker^11^. Links to both RACE-PRO and the PRO tracker can be found on the PRO home page.

The RACE-PRO entries were checked by a PRO editor and converted to PRO terms using a semi-automated process, in which standard names and definitions for gene level and isoform level terms are automatically generated as are missing parent terms that are necessary to complete the PRO hierarchy. Definitions of modified protein forms and PRO terms for complexes and families were handled manually. The end result of the processing pipeline were OBO stanzas containing the term IDs, names, definitions, synonyms, categories, and relationships to other terms. Annotations were included in the PRO Annotation File (PAF). All terms and annotations generated in this study can be found in PRO release 32.

### Analysis and visualization of the PRO terms

Once data was entered into the PRO framework, it was analyzed and visualized using the search and graphical display tools in the PRO website. The search functionality allows all parts of a PRO entry, including definition and annotation, to be searched. Query terms can be words or phrases or unique identifiers from other resources such as Pfam or GO. Searches can be restricted to a particular field of a PRO entry; for example, searching for the term “9606” in the Taxon ID field will retrieve all human protein terms. The search terms “NOT NULL” and “NULL” can be used to identify PRO entries that do or do not contain information in a selected field. Multiple search terms can be joined with the Boolean terms “AND,” “OR,” and “NOT” to carry out more complex searches. In addition, searches can be restricted to particular categories of PRO entries such as modified forms, disease-related forms, or complexes using the “Quick Links” menu provided on the PRO search page. Finally, the search result table can be customized to include/remove information and can be downloaded in tab-delimited format.

The PRO hierarchy can be visualized using a built-in tool based on Cytoscape Web (Lopes et al., 2010). The tool can be accessed by clicking on the “Cytoscape view” icon on any PRO entry page. The display can be set to show the parent(s), siblings, and/or children of the entry with or without organism-specific terms. Either sequence level or modification-level child terms can be viewed. Advanced display options allow the user to show or hide nodes based on their PRO Category (e.g., “organism-gene” or “complex”) and to hide individual nodes of choice. Selecting any node in the display provides the option to jump to the Cytoscape web view, PRO entry page, or text-based hierarchy for that node. Using the batch entry mode, the user can add terms to the display by entering their PRO or GO IDs as a comma separated list. A feature that displays the Cytoscape Web view of multiple terms selected from the PRO search results page will be available soon.

### Analysis of PRO data with external tools

The kinetochore protein–protein interaction (PPI) network was displayed using locally installed Cytoscape, version 2.8 (Smoot et al., 2011). To construct the network, we first searched PRO for all terms annotated with kinetochore or centromere localization using the query: “Taxon ID 9606 (human) AND Ontology ID GO:0000776 (kinetochore) OR Taxon ID 9606 (human) AND Ontology ID GO:0000779 (condensed chromosome, centromeric region),” and downloaded the OBO stanzas and PAF for the 34 search results. Using a script (available upon request), we extracted the name, definition, category, and label (PRO-short-label) from the OBO stanzas as well as parent-child and kinase-substrate relationships. Parent-child relationships (identified by the “is_a” relation) were directly extracted from the PRO terms. Kinase information appears in the free-text comment field of the OBO stanza; however, it could be parsed out because it is entered by PRO curators in a standardized format (Kinase = “name”; PRO ID). Protein binding related annotations (identified by the GO evidence code “inferred from physical interaction” or IPI) were extracted from the PAF. The script then generated two tab-delimitated text files, which are importable into Cytoscape: a network file containing each pair of interacting proteins, its interaction type, and corresponding evidence and a PRO entry information file containing PRO ID and entity description. Those two files were further converted into visualized protein networks with the Cytoscape functions “Import → Network from table” and “Import → Attribute from table” functions. In these networks, each node is a PRO entry and two nodes were connected by an edge if they were associated by a relation. Entity descriptions and relations annotations were represented as node or edge attributes.

Multiple sequence alignments were performed using ClustalW version 2.1 (Larkin et al., 2007; Goujon et al., 2010) and visualized with Jalview Desktop version 2.8 (Waterhouse et al., 2009). Experimentally determined phosphorylation sites taken from PRO phosphorylation site data and phosphorylation sites predicted based on sequence alignment were highlighted in the Jalview display.

## Results

### Overview of the PRO representation of the spindle checkpoint

**Figure 1:**
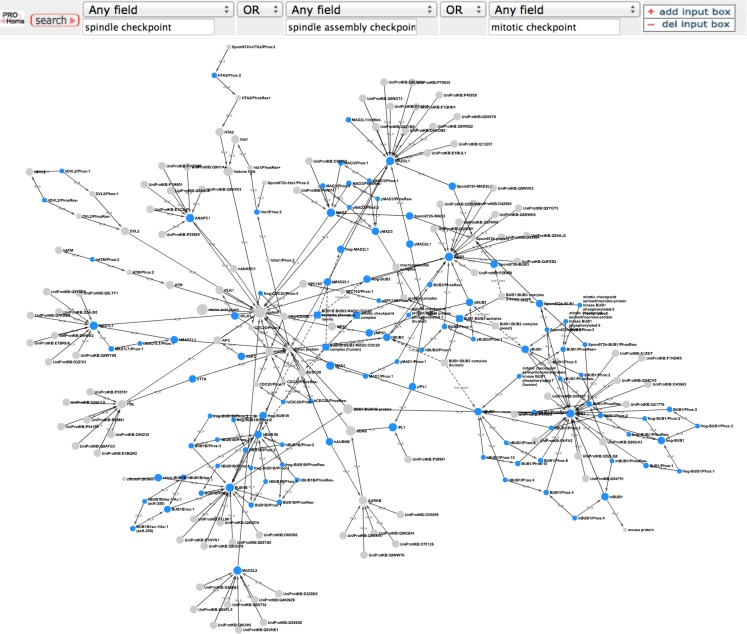
**Overview of the PRO representation of the spindle checkpoint**. PRO search query to retrieve PRO terms that contain the phrases “spindle checkpoint” or “spindle assembly checkpoint” or “mitotic checkpoint” and combined Cytoscape web view of the search results. In the Cytoscape view, nodes retrieved by the search are blue; related nodes (parents and children) are gray. Circles represent proteins or protein forms; squares represent protein complexes.

To get an overview of the extent of spindle checkpoint-related information contained within PRO we performed a search in PRO for terms containing the phrases “spindle checkpoint,” “spindle assembly checkpoint,” or “mitotic checkpoint.” The search returned 112 PRO terms. The PRO search query and the Cytoscape web view of the combined hierarchy of the search result terms are shown in Figure 1. The hierarchy, which includes parents and children of the search result terms as well as complexes containing the search result terms, consists of 208 terms (including two obsolete terms) spanning all levels in PRO. There are three family level terms – Histone H2A (PR:000027547), Aurora Kinase (PR:000035365), and BUB1/BUB1B (PR:000035665) – and 21 gene-level terms, including the seven core checkpoint proteins. Of the 35 modification-level terms, 26 are phosphorylated forms, 6 are unphosphorylated forms, 1 is an acetylated form, and 1 is a cleaved form. The figure also includes one sequence level term [BUB1B isoform 1 (PR:000028795)]; two complexes [BUB1:BUB3 complex (PR:000035566) and the mitotic checkpoint complex (MCC; GO:0033597)]; and the high level terms amino acid chain (PR:000018263), protein (PR:000000001), macromolecular complex (GO:0032991), and protein complex (GO:0043234). The 140 organism-specific terms (75 organism-gene, 47 organism-modification, 1 organism-sequence, and 17 organism-complex terms) span a wide evolutionary range, including terms from humans, rodents, frogs, plants, insects, worms, and yeast. We will consider some specific questions that can be addressed by this representation in the sections that follow.

### Evolutionary relationship of BUB1, BUB1B, and MAD3

The spindle checkpoint pathway is highly conserved throughout eukaryotes. Homologs of the core checkpoint proteins are present in organisms from yeast to humans and checkpoint mechanisms, such as MCC inhibition of the APC/C, are also conserved (Zich and Hardwick, 2010; Lara-Gonzalez et al., 2012). Despite the overall similarity, there are significant differences in the details of the sequence and function of some the checkpoint proteins. One of the most striking examples of this variation involves the “BUB-like” proteins, BUB1, BUB1B, and MAD3. Derived from a common ancestor, modern BUB-like proteins arose as the result of multiple gene duplication events. Some organisms have only one of these proteins; others, like *Arabidopsis thaliana*, have as many as three (Suijkerbuijk et al., 2012). Humans have two (BUB1 and BUB1B). Budding and fission yeasts also have two: BUB1, which is orthologous to human BUB1, and MAD3, which is most closely related to human BUB1B. BUB1, BUB1B, and MAD3 share an N-terminal domain containing tetratricopeptide repeats [TPR domain; (D’Arcy et al., 2010)]. This domain of budding yeast MAD3 has been shown to bind to the APC/C subunit, CDC20, an interaction critical for checkpoint-mediated inhibition of anaphase onset (Hardwick et al., 2000). Outside of this N-terminal region, however, BUB1, BUB1B, and MAD3 diverge significantly. BUB1 and BUB1B contain a C-terminal kinase domain, which is absent from MAD3. BUB1 is a bona fide protein kinase, whereas BUB1B is likely to be a pseudokinase, although BUB1B kinase activity, particularly auto-phosphorylation activity under some conditions, remains a possibility (Guo et al., 2012; Suijkerbuijk et al., 2012).

#### What can we learn about the evolutionary relationship of BUB1, BUB1B, and MAD3 using the PRO website?

In PRO, ProEvo classes provide insight into the evolutionary relationships among proteins by grouping proteins that share full-length sequence similarity. Importantly, this higher level relationship based on a common domain organization can be searched in PRO, as terms in ProEvo are annotated with domain information from resources such as Pfam. Therefore, we searched PRO for proteins that contained the conserved N-terminal TPR domain found in all of the BUB-like proteins (PFAM:PF08311, MAD3/Bub1 homology domain I). The search returned two results: the MAD3 gene-level term (PR:000035499) and the BUB1/BUB1B family level term (PR:000035665).

**Figure 2:**
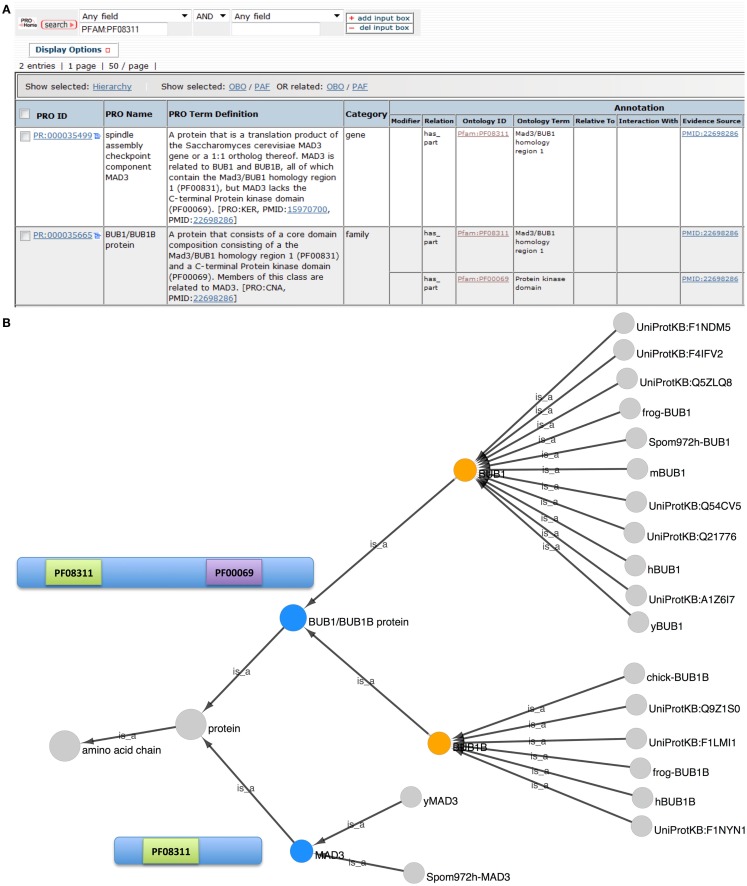
**Evolutionary relationship of BUB1, BUB1B, and MAD3**. **(A)** PRO search results page showing the terms retrieved in a search for “PFAM:PF08311.” Fields shown in the display were set using “Display Options.” **(B)** Combined Cytoscape web view of the BUB1/BUB1B family term (PR:000035665) and the MAD3 gene-level term (PR:000035499) (blue nodes). Advanced display options were set to include family, gene, and gene-organism terms only. The BUB1 and BUB1B gene-level nodes are orange. Diagrams of the MAD3 and BUB1/BUB1B proteins show the location of Pfam domains PF08311 (MAD3/Bub1 homology domain I) and PF00069 (protein kinase domain).

To reveal the common and divergent attributes of these protein classes, the result table was customized, via the Display Option functionality, to display the corresponding annotations and allow their direct comparison **(**Figure 2A**)**. As expected both groups are annotated as containing the MAD3/Bub1 homology domain I (PFAM:PF08311), and the definition of the BUB1/BUB1B family states in part that: “Members of this class are related to MAD3.” However, the BUB1/BUB1B proteins contain a second conserved domain, the C-terminal protein kinase domain (PFAM:PF00069) that is absent in the MAD3 class.

The combined Cytoscape web view for BUB1/BUB1B and MAD3 terms is shown in Figure 2B. BUB1/BUB1B and MAD3 (blue nodes) are connected by the parent term “protein.” BUB1 (PR:000004854) and BUB1B (PR:000004855) are both children of the BUB1/BUB1B class, indicating that these two proteins share full-length sequence similarity. BUB1 is very highly conserved with 11 organism-specific child terms ranging from yeast to human. Compared to BUB1, BUB1B is less conserved. Its children include human and frog BUB1B terms but no yeast terms. Instead, the closest yeast relative of BUB1B is MAD3 (PR:000035499).

### Prediction of BUB1B phosphorylation sites

Phosphorylation is a major mechanism of regulation in the spindle checkpoint pathway and the interplay among the checkpoint-related phosphorylation events is complex (Zich and Hardwick, 2010). There are multiple spindle checkpoint kinases, each of which has multiple substrates. Some checkpoint proteins are targeted by more than one kinase and exist in several phosphorylated forms. One such protein, BUB1B, has at least four different mitotic phosphorylated forms (Elowe et al., 2007, 2010; Matsumura et al., 2007; Wong and Fang, 2007; Huang et al., 2008; Guo et al., 2012). Phosphorylated forms of BUB1B first appear during pro-metaphase as condensed chromosomes begin to make attachments to spindle microtubules and persist until all chromosomes have made correct bipolar attachments to the spindle at metaphase. Although BUB1B was first characterized as a spindle checkpoint protein, phosphorylated forms of BUB1B have been shown to participate in spindle assembly as well.

#### How can we look at the different phosphorylated forms of BUB1B in PRO? Are these forms conserved and what predictions can we make?

**Figure 3:**
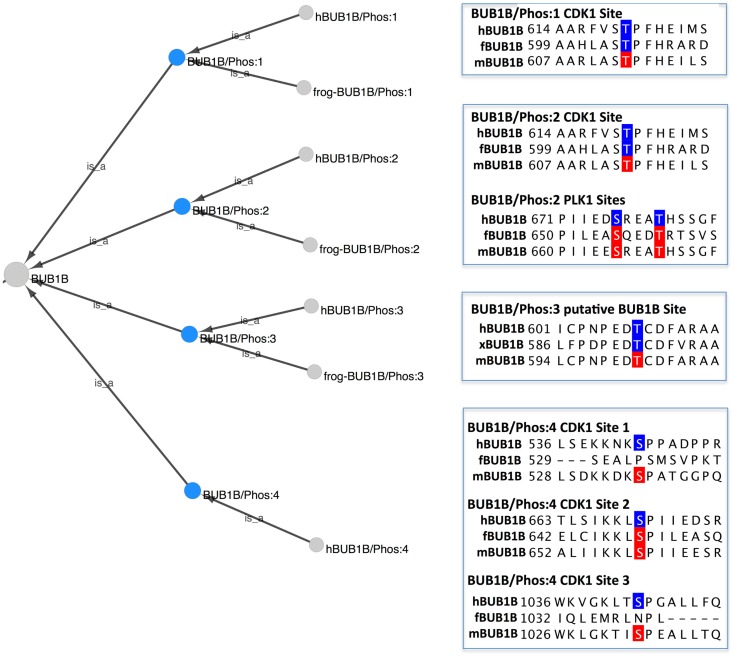
**Phosphorylated forms of BUB1B**. Combined Cytoscape web view of the four species-independent BUB1B phosphorylated forms (blue nodes): BUB1B/Phos:1 (PR:000035361), BUB1B/Phos:2 (PR:000035427), BUB1B/Phos:3 (PR:000035431), and BUB1B/Phos:4 (PR:000035434). Display options were set to show parents and all children, including organism level terms. Portions of a sequence alignment of human, frog, and mouse BUB1B are highlighted to indicate experimentally determined phosphorylation sites (blue) and predicted phosphorylation sites (red).

To view the phosphorylated BUB1B protein forms in PRO, we searched for “bub1 beta” in the PRO Name field, restricting the search to phosphorylated forms using the Quick Links menu. Eleven search results were returned: four species-independent modification-level terms and seven species-specific terms. The combined Cytoscape web view of the four species-independent terms (PR:000035361, PR:000035427, PR:000035431, and PR:000035434) is shown in Figure 3. The four phosphorylated forms have the species-independent BUB1B gene-level term as their common parent. Each form also has one or more organism-specific children. Alongside each form is a portion of a sequence alignment of human, frog, and mouse BUB1B with experimentally confirmed form-specific phosphorylation sites highlighted in blue and predicted phosphorylation sites highlighted in red.

BUB1B/Phos:1 (PR:000035361), defined in PRO as a BUB1B form that has been phosphorylated on a site analogous to Thr-620 of human BUB1B, is found in humans (PR:000035362) and frogs (PR:000035426). The frog form is phosphorylated on Thr-605, which is considered to be analogous to human Thr-620 because it aligns with human Thr-620 in a multiple sequence alignment (Figure 3, BUB1B/Phos:1, blue residues). In both organisms, the phosphorylation is carried out by the cyclin-dependent kinase CDK1 (see PRO entry pages, comment section).

Although BUB1B/Phos:1 has not as yet been characterized in mice, the equivalent phosphorylation site (Thr-613) is conserved in the mouse protein (Figure 3, BUB1B/Phos:1, red residue). Furthermore, Thr-613 of mouse BUB1B was identified as an *in vivo* phosphorylation site in a high throughput study of mitotic phosphorylation (Hegemann et al., 2011). Thus, there is a high probability that BUB1B/Phos:1 exists in mice as well.

BUB1B/Phos:2 (PR:000035427) contains the same CDK1 phosphorylation site (Thr-620 in humans) as BUB1B/Phos:1 and is additionally phosphorylated on several sites by PLK1/PLX1. Because experimental evidence indicates that PLK1 phosphorylation of BUB1B is low in the absence of prior CDK1 phosphorylation, PRO does not have a term for BUB1B phosphorylated by PLK1 alone (Elowe et al., 2007; Wong and Fang, 2007). As described in its PRO definition, human BUB1B/Phos:2 (PR:000035428) is observed during pro-metaphase when kinetochores are undergoing attachment to the mitotic spindle and under conditions that depolymerize the spindle (nocodazole treatment) or that disrupt the ability of microtubules to apply tension across kinetochores (taxol treatment).

The PLK1 phosphorylation sites in BUB1B/Phos:2 are a subject of ongoing investigation. The PRO entry page for the human BUB1B/Phos:2 (PR:000035428) documents two neighboring sites – Ser-676 and Thr-680 – that have been verified *in vivo* and two other sites – Thr-792 and Thr-1008 – that have so far only been observed in *in vitro* studies. The *in vivo* sites are shown in the sequence alignment in Figure 3 (BUB1B/Phos:2 PLK1 sites, blue residues).

One of the challenging aspects of the curation of PRO phosphorylated forms is determining whether a phosphorylated form that has been defined in one species also exists in other species. This challenge is exemplified by BUB1B/Phos:2. There is evidence that BUB1B/Phos:2 exists in both frogs and mice, although it has not been completely characterized in either organism. All of the human BUB1B/Phos:2 phosphorylation sites that have been confirmed *in vivo* are conserved in the frog and mouse proteins (frog: Thr-605, Ser-655, and Thr-659; mouse: Thr-613, Ser-665, and Thr-669; Figure 3). Moreover, a phosphorylated form of BUB1B has been observed in frogs and mice in the same conditions – the presence of unattached kinetochores – under which BUB1B/Phos:2 is observed in humans (Taylor et al., 2001; Chen, 2002). This evidence alone was determined to be insufficient to create a PRO term; however, in frogs there is additional evidence in support of the existence of BUB1B/Phos:2. First, frog BUB1B is known to be phosphorylated by CDK1 on Thr-605; this phosphorylation is analogous to the CDK1 phosphorylation site in human BUB1B/Phos:2 (Thr-620). Second, as is the case in humans, CDK1 phosphorylation of frog BUB1B at Thr-605 stimulates the further phosphorylation of BUB1B by frog PLK1 (Wong and Fang, 2007). Thus, a PRO term was created for frog BUB1B/Phos:2 (PR:000035430). We predict that BUB1B/Phos:2 is also present in mice, but more experimental work is necessary to demonstrate its existence.

BUB1B/Phos:3 (PR:000035431) is phosphorylated on Thr-608 in humans (PR:000035432) and on the equivalent site, Thr-593 in frog (PR:000035433) (Figure 3; BUB1B/Phos:3, blue residues). An analog of BUB1B/Phos:3 has not been characterized in mice, but the phosphorylation site is conserved (mouse Thr-601; Figure 3; BUB1B/Phos:3, red residue) and has been shown to be phosphorylated *in vivo* (Hegemann et al., 2011). The proposed kinase for BUB1B/Phos:3 is BUB1B itself in association with the kinetochore component, CENPE (Guo et al., 2012). However, a recent structural and functional analysis indicates that BUB1B does not have kinase activity, but is instead a pseudokinase (Suijkerbuijk et al., 2012). To reflect this uncertainty, the comment section of the PRO record for the frog and human BUB1B/Phos:3 PRO entry pages states: “One of the articles cited mentions BUBR1 (PR:000026903) as the kinase when bound to CENPE (PR:000035367).”

Finally, BUB1B/Phos:4 (PR:000035435), which has so far only been observed in humans (PR:000035435), is multiply phosphorylated by CDK1 on sites distinct from those phosphorylated in BUB1B/Phos:1 and BUB1B/Phos:2. Phosphorylation occurs *in vivo* on at least three CDK1 consensus sites: Ser-543, Ser-670, and Ser-1043 (see PR:000035435, term definition). All three sites are conserved in mouse BUB1B and two of the three (mouse Ser-535 and Ser-1033) have been shown to be phosphorylated *in vivo*, strongly suggesting that BUB1B/Phos:4 exists in mouse [Figure 3; BUB1B/Phos:4, red residues; (Hegemann et al., 2011)]. BUB1B/Phos:4 does not exist in frogs because only one of the phosphorylation sites (human Ser-670, frog Ser-649) is conserved (Figure 3; BUB1B/Phos:4, red residues). However, it is noteworthy that mutation of Ser-670 alone in the human BUB1B protein produced phenotypes nearly as severe as mutating all of the BUB1B/Phos:4 sites, indicating that Ser-670 is a critical phosphorylation site (Huang et al., 2008; Elowe et al., 2010). Thus, it is possible that frog has a BUB1B form phosphorylated on Ser-649 that plays a similar role to BUB1B/Phos:4 in humans.

By combining the PRO representation of phosphorylated forms with multiple sequence alignments, we can predict not just individual phosphorylation sites, but combinations of phosphorylation sites that are likely to occur *in vivo*. Thus, we predict that mice will have a BUB1B/Phos:1 (phosphorylated on Thr-613), a BUB1B/Phos:2 (phosphorylated on Thr-613, Ser-665 and Thr-669), a BUB1B/Phos:3 (phosphorylated on Thr-601), and a BUB1B/Phos:4 (phosphorylated on mouse Ser-535, Ser-559, and Ser-1033). Frogs probably have a BUB1B/Phos:2 (phosphorylated on Thr-605, Ser-655, and Thr-659). Due to lack of phosphorylation site conservation, frogs cannot have a BUB1B/Phos:4. It would be interesting to investigate whether this difference in BUB1B phosphorylation has any biological implications.

### Analysis of spindle checkpoint protein complexes

In the presence of unattached or incorrectly attached kinetochores, the core spindle checkpoint proteins form multiple protein complexes that contribute to the inhibition of the APC/C and metaphase arrest (Zich and Hardwick, 2010). Representation of these complexes in PRO facilitates comparisons of complex composition and the conservation of complexes across organisms. In this study, we used PRO to address questions about the APC/C inhibitory MCC and complexes containing the checkpoint kinase BUB1.

#### What is the function and subunit composition of the MCC?

**Figure 4:**
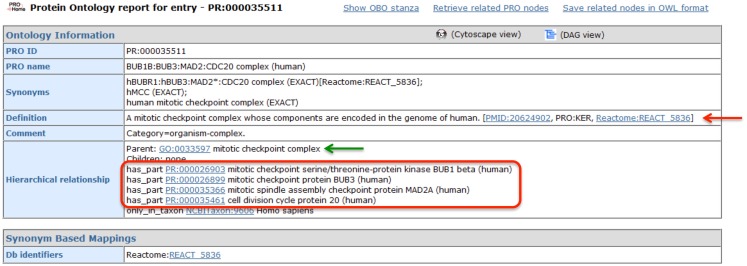
**PRO entry page for the human MCC**. Screenshot of the PRO entry page for the human MCC (PR:000035511). Complex components are indicated by the red circle. Links to GO and Reactome are indicated by green and red arrows, respectively.

The MCC is one of the best-characterized spindle checkpoint complexes, and consequently, it has been described in multiple bioinformatics resources, including GO and Reactome. The PRO record for the human MCC (PR:000035511), shown in Figure 4, demonstrates how PRO interoperates with these other resources, augmenting the representation of the complex without unnecessarily duplicating information. First, GO provides the species-independent parent term for the complex (GO:0033597; green arrow). The GO record includes the following definition of the MCC that describes its function and composition: “A multi-protein complex that functions as a mitotic checkpoint inhibitor of the anaphase-promoting complex/cyclosome (APC/C). In budding yeast this complex consists of Mad2p, Mad3p, Bub3p, and Cdc20p, and in mammalian cells it consists of MAD2, BUBR1, BUB3, and CDC20.” Complex component information is also provided by Reactome (REACT 5836; red arrow). In the PRO record, the complex components are listed in the “Hierarchical Relationship” section associated with the ontological relation “has_part” (red box). Thus, PRO provides an ontological representation of the human MCC that brings together the GO definition of the complex with species-specific component information from Reactome.

#### Are BUB1-containing complexes conserved across species?

The BUB1 protein plays a critical role in checkpoint signal generation. Together with BUB3, it localizes to kinetochores by binding to the kinetochore component CASC5 (KNL1/blinkin) and serves as a platform for the recruitment and activation of other checkpoint proteins, including MAD1 and BUB1B (Lara-Gonzalez et al., 2012).

**Figure 5:**
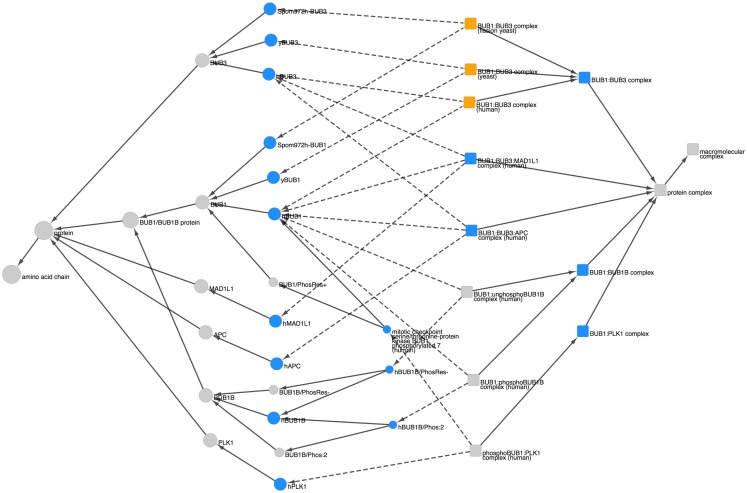
**BUB1-containing complexes**. Combined Cytoscape web view of BUB1-containing complexes (squares) and their components (circles). The following terms were used to generate the display: complex terms (blue squares): BUB1:BUB3 (PR:000035566), BUB1:BUB3:MAD1L1 (PR:000035567), BUB1:BUB3:APC (PR:000035576), BUB1:BUB1B (PR:000035578), and BUB1:PLK1 (PR:000035580). Component terms (blue circles): hBUB1 (PR:000035400), hBUB3 (PR:000026899), yBUB1 (PR:000035402), yBUB3 (PR:000035532), SpomBUB1 (PR:000035570), SpomBUB3 (PR:000035571), hMAD1L1 (PR:000035474), hAPC (PR:000030190), hBUB1B (PR:000026903), hBUB1B/Phos:2 (PR:000035428), hBUB1B/PhosRes- (PR:000035373), hBUB1/Phos:7 (PR:000035412), and hPLK1 (PR:000035455). Species-specific BUB1:BUB3 complexes are shown in orange. Dotted arrows indicate the has_part relation; solid arrows indicate the is_a relation. Display options were set to show parents and all children, including organism level terms; nodes for siblings of complex components and complexes not containing BUB1 were hidden.

To view the PRO representation of BUB1-containing complexes, we searched for “BUB1” in any field and restricted the search results to complexes using the Quick Links menu. The search returned 16 results, including 11 BUB1 complexes (The other five complexes contained BUB1B rather than BUB1.). The combined Cytoscape web view of these 11 complexes and their components is shown in Figure 5. The complex terms (squares) and component terms (circles) that were used to generate the display are shown in blue.

BUB1 and BUB3 appear together in three different complexes: BUB1:BUB3 (PR:000035566), BUB1:BUB3:MAD1L1 (PR:000035567), and BUB1:BUB3:APC (PR:000035576) [Note: APC is the short name for the adenomatous polyposis coli protein (APC); it is not the anaphase-promoting complex/cyclosome (APC/C).]. The BUB1:BUB3 complex is highly conserved, occurring in human, fission yeast, and budding yeast (orange squares). The BUB1 proteins from all three organisms have a common parent (the species-independent BUB1 term, PR:000004854), indicating that they are orthologous; similarly, the BUB3 proteins have the species-independent BUB3 term (PR:000004856) as a common parent. Given that orthologous BUB1:BUB3 complexes exist in distantly related organisms (humans and yeast) we expect that more examples of this complex will be added to PRO in the future as more of the spindle checkpoint literature is curated. The BUB1:BUB3:MAD1L1 complex, so far observed only in humans, forms at kinetochores during the process of checkpoint activation (Seeley et al., 1999). Although the function of the BUB1:BUB3:APC complex is not known, it is interesting to note that APC, a microtubule-binding protein found at kinetochores, is phosphorylated by BUB1:BUB3 [see PRO annotation for APC (PR:000030190) and APC/Phos:1 (PR:000030182)].

The remaining BUB1-containing complexes in Figure 5, BUB1:BUB1B and BUB1:PLK1, illustrate the ability of PRO to represent information about the modification state of complex components. In the case of the BUB1:BUB1B complex, BUB1 can bind to unphosphorylated BUB1B, but complex formation is enhanced by the mitotic phosphorylation of BUB1B (Taylor et al., 2001). Thus, there are two human BUB1:BUB1B complexes in PRO: one consists of BUB1 and the unphosphorylated form of BUB1B (PR:000035579) and the other consists of BUB1 and BUB1B/Phos:2 (PR:000035577). The two complexes have the subunit BUB1 in common but contain different forms of BUB1B. Both complexes are children of the species-independent BUB1:BUB1B complex (PR:000035578). In the case of the BUB1:PLK1 complex, phosphorylation of human BUB1 on Ser-593 and Thr-609 by CDK1 is required for its binding to the polo-like kinase, PLK1, and for the recruitment of PLK1 to kinetochores (Qi et al., 2006). Thus, the BUB1-PLK1 complex term in PRO (PR:000035580) has only one child, the phosphoBUB1:PLK1 complex (PR:000035581) that consists of PLK1 and the CDK1-phosphorylated form of BUB1, BUB1/Phos:7.

### A protein interaction network for spindle checkpoint proteins at the kinetochore

The kinetochore, a complex, multi-protein structure organized around the centromeric DNA of each sister chromatid pair, is critically important as a staging area for the generation and amplification of spindle checkpoint signals (Lara-Gonzalez et al., 2012). In addition to its role in the spindle checkpoint, the kinetochore has other vital functions, including spindle microtubule binding and regulation of sister chromatid cohesion (Hori and Fukagawa, 2012). Using information downloaded from PRO, we created a network that illustrates the PPIs between checkpoint proteins and other proteins that reside at the kinetochore.

#### What are the PPIs observed between checkpoint proteins and other proteins in the kinetochore?

**Figure 6:**
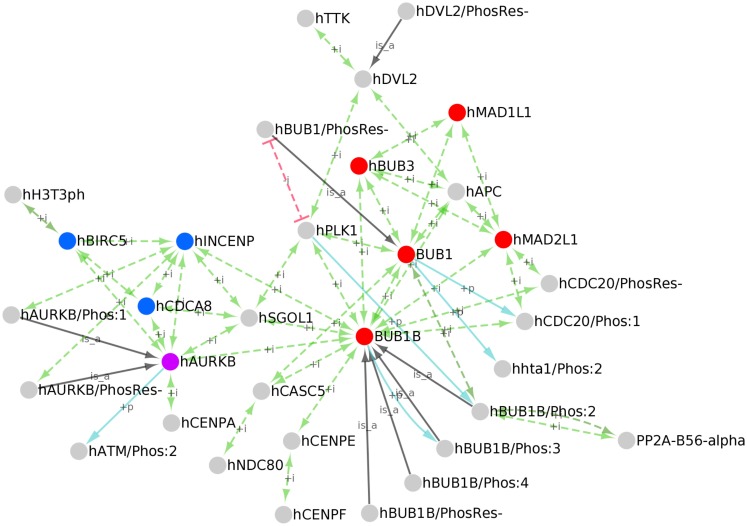
**PPI network of kinetochore/centromere localized proteins**. Cytoscape network of the kinetochore/centromere localized proteins in PRO. PPIs (green edges), inhibited PPIs (red edges), kinase/phosphorylated product relationships (blue edges), and parent-child relationships for phosphorylated forms (black edges) are shown. Nodes representing the core spindle checkpoint proteins BUB1, BUB1B, BUB3, MAD1, and MAD2 are red; nodes representing the Chromosomal Passenger Complex (CPC) subunits INCENP, CDCA8, and BIRC5 are blue; AURKB is purple.

To create a PPI network of kinetochore-localized proteins, we first identified all human kinetochore-localized protein forms in PRO by searching for terms with Taxon ID 9606 (human) and Ontology ID GO:0000776 (kinetochore). Although the kinetochore and centromere are distinct structures, the terms are sometimes used interchangeably in the literature; therefore, we also retrieved human centromere localized proteins by searching for human proteins (Taxon ID 9606) annotated with the GO term GO:0000779 (condensed chromosome, centromeric region). The searches returned 34 results, including 28 kinetochore-localized protein forms, 5 centromere localized forms, and one term – AURKB (PR:000035358) that is annotated with both kinetochore and centromere localization terms. These terms are annotated with PPI data mined from the literature and from several PPI databases. We downloaded the OBO stanzas and PAF for these proteins from PRO and used the information therein to build a network with Cytoscape (Figure 6). In addition to the PPIs (green arrows), the network displays kinases for the phosphorylated protein forms (blue arrows) and gene-level parent terms for the modification-level terms (black arrows).

Because functional annotation of PRO terms is an ongoing process, the set of kinetochore/centromere localized proteins we retrieved is not comprehensive nor is the PRO annotation of PPIs for these proteins complete. However, it is representative of the diverse functions of the kinetochore. The core checkpoint proteins BUB1, BUB1B, BUB3, MAD1L1, and MAD2L1 (Figure 6, red nodes) are found at the kinetochore/centromere and interact extensively with each other. All of the possible pair-wise interactions among these proteins are present except for BUB1B-MAD1 and BUB1-MAD2. The core checkpoint protein AURKB (purple) associates with this sub-network via an association with BUB1B. The checkpoint target CDC20 is also found at kinetochores/centromeres where it interacts with the MCC components MAD2, BUB1B, and BUB3. BUB1-dependent phosphorylation of CDC20 does not affect its ability to bind other MCC components as both CDC20/Phos:1 and CDC20/PhosRes-interact with MAD2 and BUB1B.

The checkpoint proteins are integrated into the larger environment of the kinetochore through interactions with other kinetochore/centromere proteins. AURKB binds to BIRC5, CDCA8, and INCENP (Figure 6, blue nodes) to form the Chromosomal Passenger Complex (CPC; van der Waal et al., 2012). Both phosphorylated (AURKB/Phos:1) and unphosphorylated (AURKB/PhosRes-) forms interact with INCENP, suggesting that AURKB phosphorylation does not play a role in CPC formation. Several CPC subunits (AURKB, INCENP, and CDCA8) interact with SGOL1, a protein that participates in sister chromatid cohesion [see PRO annotation for SGOL1 (PR:000035551)]. The CPC is tethered to the centromere via interactions with centromeric histone subunits. In particular, the CPC subunit AURKB interacts with the centromeric histone H3 variant CENPA and BIRC5 interacts with the Thr-3 phosphorylated form of histone H3 (H3T3ph).

BUB1 and BUB1B both associate with the outer kinetochore component, CASC5. BUB1B makes other connections to the kinetochore via SGOL1 and CENPE, a protein that assists in the alignment of chromosomes on the metaphase plate [see PRO annotation for CENPE (PR:000035367)]. BUB1B binding to CENPE may stimulate its auto-phosphorylation activity [see BUB1B/Phos:3 (PR:000035432)].

Several spindle checkpoint proteins – BUB1, BUB1B, BUB3, and MAD2 – interact with APC and the checkpoint kinase TTK interacts with DVL2. APC and DVL2, which interact with each other, both participate in spindle assembly [see PRO annotation for APC (PR:000030190) and DVL2 (PR:000035487)]. The significance of these interactions is unclear, but it could reflect a role for APC and DVL2 in checkpoint signaling or a role for the checkpoint proteins in spindle assembly.

A protein kinase, PLK1, and a protein phosphatase, PP2A, associate with checkpoint proteins and other kinetochore proteins, positioning them to regulate critical kinetochore substrates. PLK1 interacts with the checkpoint proteins BUB1 and BUB1B as well as SGOL1 and DVL2. PLK1 association with BUB1 depends upon the prior phosphorylation of BUB1 (Qi et al., 2006); this dependence is represented in the network by a red line indicating an inhibited interaction between unphosphorylated BUB1 (BUB1/PhosRes-) and PLK1. As previously discussed, PLK1 phosphorylates BUB1B [see BUB1B/Phos:2 (PR:000035428)]. Additional kinetochore-localized substrates of PLK1 most likely exist. For example, human BUB1 is phosphorylated by CDK1 and PLK1 in a manner analogous to BUB1B/Phos:2 (BUB1/Phos:8; PR:000035418); however, further experiments are necessary to show that this form is indeed localized to kinetochores and to determine its role in spindle assembly and checkpoint function. The phosphatase PP2A localizes to kinetochores by binding to phosphorylated BUB1B (BUB1B/Phos:2).

### The role of protein phosphorylation at the kinetochore/centromere

Eight of the kinetochore/centromere localized protein forms in our set are phosphorylated: BUB1B/Phos:2, BUB1B/Phos:3, BUB1B/Phos:4, AURKB/Phos:1, CDC20/Phos:1, ATM/Phos:2, H3T3ph, and HHTA1/Phos:2. Because phosphorylation can have a wide range of effects on proteins, affecting localization, function, and/or the processes in which they participate, we wanted to investigate the impact of phosphorylation on these particular proteins.

#### What functions, processes, and subcellular localizations are affected by protein phosphorylation in the human kinetochore?

In the PRO annotation, localizations, functions, and processes that are affected by protein modification are denoted by adding a modifier (such as increased or decreased) to the corresponding GO term and inclusion of a reference form. Thus, we searched for human proteins (Taxon ID 9606) localized to the kinetochore (Ontology ID GO:0000776) with at least one line of functional annotation that included a modifier (Modifer NOT NULL); to limit the results to phosphorylated proteins, we selected “Phosphorylated forms” from the Quick Links menu. For the reasons described above, we repeated the search substituting GO:0000779 (condensed chromosome, centromeric region) in the Ontology ID field. All eight of the kinetochore/centromere localized proteins appeared in our search results, indicating that all of these proteins had at least one attribute that was affected by phosphorylation. We examined the annotation for each protein and summarized the affected attributes in Table 1.

**Table 1:** **Functional effects of phosphorylation of kinetochore/centromere localized proteins**.

Protein	Modifier	Function/process	Targets
CDC20/Phos:1	Decreased	Ubiquitin protein ligase activity	
	Increased	Spindle checkpoint	
BUB1B/Phos:2	Increased	Protein binding	BUB1
	Increased	Protein binding	PP2A
	Increased	Protein kinase activity	
	Increased	Attachment of spindle microtubules to kinetochores	
	Increased	Metaphase plate congression	
BUB1B/Phos:3	Increased	Metaphase plate congression	
	Increased	Chromosome segregation	
	Increased	Spindle checkpoint	
	Increased	Protein localization to kinetochore	MAD1L1, MAD2L1
	Decreased	Negative regulation of protein phosphorylation	NDC80
BUB1B/Phos:4	Increased	Attachment of spindle microtubules to kinetochores	
	Increased	Inhibition of mitotic anaphase-promoting complex activity	
	Increased	Metaphase plate congression	
AURKB/Phos:1	Increased	Protein kinase activity	
	Increased	Chromosome segregation	
	Increased	Metaphase plate congression	
	Increased	Spindle checkpoint	
ATM/Phos:2	Increased	Protein kinase activity	
	Increased	Spindle Checkpoint	
HHTA1/Phos:2	Increased	Protein localization to chromosome, centromeric region	SGOL1
H3T3ph	Increased	Protein binding	BIRC5
	Increased	Protein localization to chromosome, centromeric region	AURKB, CDCA8, INCENP, BIRC5

*PRO terms retrieved using the search query: “Taxon ID 9606 (human) AND Ontology ID GO:0000776 (kinetochore) AND Modifier NOT NULL OR Taxon ID 9606 (human) and Ontology ID GO:0000779 (condensed chromosome, centromeric region) and Modifier NOT NULL.” Results were restricted to phosphorylated proteins only by selecting “Phosphorylated forms” from the Quick Links menu. Protein binding partners were obtained from the “Interaction with” column of the Functional Annotation section of the PRO entry page. Targets for the annotation terms “Protein localization to the kinetochore,” “Protein localization to the chromosome, centromeric region,” and “Negative regulation of protein phosphorylation” were obtained from the comment column of the PAF*.

Even though phosphorylation is often used as a mechanism to regulate protein localization, none of the phosphorylated proteins in this group was annotated to indicate increased or decreased localization to the kinetochore/centromere relative the unphosphorylated form. In fact, the unphosphorylated forms of several of these proteins – BUB1B, CDC20, and AURKB – have been shown to localize to kinetochores with similar affinity as the phosphorylated forms see PRO annotation for BUB1B/PhosRes-(PR:000035373), CDC20/PhosRes-(PR:000035369), and AURKB/PhosRes-(PR:000035661). Intriguingly, the kinases for CDC20/Phos:1 (kinase is BUB1), BUB1B/Phos:2 (kinase is PLK1), ATM/Phos:2 (kinase is AURKB), and HHTA1/Phos:2 (kinase is BUB1), are themselves kinetochore/centromere localized proteins (see Figure 6). In addition, BUB1B/Phos:3 phosphorylation depends on the association of BUB1B with the kinetochore-localized protein, CENPE. Taken together, these observations suggest that phosphorylation may occur after kinetochore localization. It would be interesting to test this hypothesis and to see if it holds true for a wider range of phosphorylated kinetochore/centromere localized proteins.

While phosphorylation did not affect the ability of these proteins to localize to the kinetochore/centromere themselves, three phosphorylated protein forms (phospho-Ser-121-Histone H2A, phospho-Thr-3-Histone H3, and BUB1B/Phos:3) showed an increased ability to recruit other proteins to the kinetochore/centromere relative to their respective unphosphorylated forms. Phosphorylation of Histone H2A on Ser-121 (HHTA1/Phos:2) creates a binding site for SGOL1. Phosphorylation of Histone H3 on Thr-3 (H3T3ph) creates a binding site for BIRC5, which in turn recruits the rest of the CPC (AUKB, CDCA8, and INCENP). Finally, BUB1B/Phos:3 is required for the kinetochore recruitment of MAD1 and MAD2.

Phosphorylation of BUB1B (BUB1B/Phos:2, BUB1B/Phos:3, and BUB1B/Phos:4) and AURKB (AURKB/Phos:1) is important for the ability of these proteins to regulate microtubule/kinetochore attachments as the phosphorylated forms show increased participation in attachment of spindle microtubules to kinetochores, metaphase plate congression, and/or chromosome segregation. Formation of stable, bipolar microtubule-kinetochore attachments requires a balance of kinase and phosphatase activity. AURKB destabilizes incorrect attachments by phosphorylating kinetochore components such as NDC80; the phosphatase PP2A counterbalances AURKB activity by dephosphorylating NDC80, thereby stabilizing attachments (Zich and Hardwick, 2010; Foley et al., 2011). Because AURKB kinase activity is important for destabilizing incorrect kinetochore-microtubule attachments, the increased kinase activity of AURKB/Phos:1 may explain its enhanced role in this process (Zich and Hardwick, 2010). On the other hand, BUB1B/Phos:2 may help stabilize nascent kinetochore-microtubule attachments through its increased affinity for PP2A, the phosphatase that reverses AURKB phosphorylation of NDC80 (Foley et al., 2011). Although its interaction with PP2A has not been directly assessed, BUB1B/Phos:3 shows a decreased ability to negatively regulate NDC80 phosphorylation (i.e., NDC80 phosphorylation is increased in the presence of BUB1B/Phos:3). This suggests that BUB1B/Phos:3 might have a reduced affinity for PP2A relative to unphosphorylated BUB1B. It would be interesting to test whether BUB1B/Phos:4 also affects the NDC80 phosphorylation/dephosphorylation cycle. Overall, these results suggest that BUB1B affinity for PP2A and consequently, the stability of kinetochore-microtubule attachments may be sensitively modulated by the BUB1B phosphorylation state.

Four proteins – Cdc20/Phos:1, AURKB/Phos:1, ATM/Phos:2, and BUB1B/Phos:3 – show an increased ability to mediate the spindle checkpoint relative to their unphosphorylated counterparts. CDC20/Phos:1 (phosphorylated by BUB1) shows decreased ubiquitin ligase activity relative to unphosphorylated CDC20, which presumably leads to its increased checkpoint activity. Thus, the spindle checkpoint acts through CDC20 in two independent ways to inhibit the APC/C: through formation of the MCC (BUB1B, BUB3, MAD2, and CDC20), which binds and inhibits the APC/C, and by phosphorylation of CDC20, which inhibits its ubiquitin ligase activity. Both AURKB/Phos:1 and ATM/Phos:2 have increased protein kinase activity relative to the unphosphorylated forms, which may be important in their increased ability to participate in the checkpoint response, although this possibility has not been directly tested. BUB1B/Phos:3 may participate in the checkpoint through its recruitment of MAD1L1 and MAD2L1 to kinetochores.

## Discussion

The structural framework and features of PRO enable the investigation of many aspects of proteins and complexes, particularly analyses of cross-species relationships and relationships between modified proteins forms and functions. Our spindle checkpoint use case outlines a number of strategies that can be generalized to other cellular processes or pathways of interest.

### Investigation of the role of modified protein forms in a biological process

In this study we showed how the PRO framework could be used to investigate the role of different protein forms that participate in a biological process of interest. We focused on PTM protein forms, as PTM is a central mechanism for the regulation of protein function in cells. Most PTM resources specialize in a single type of modification (e.g., phosphorylation) and are organized around individual modification sites. However, protein modification *in vivo* is usually a combinatorial process where proteins are subject to multiple types of modifications on multiple sites. In this regard, PRO offers a more realistic view of protein modification through its representation of protein forms that carry the combinations of modifications that are observed *in vivo*. The representation of protein complexes in PRO also takes into account the modification state of the complex components. Moreover, modified forms and complexes in PRO can be individually annotated with functional information, making it possible to discern the contribution of each to a biological process.

We used PRO to explore the role of protein phosphorylation in the context of the spindle checkpoint. Our examination of the PRO representation of human BUB1B phosphorylated forms and complexes revealed multiple phosphorylated forms of this protein and at least two participating kinases (Figure 3). Comparison of the annotation of the BUB1B phosphorylated forms provided an additional level of information that revealed some intriguing phosphorylation state-dependent differences in function. For example, BUB1B/Phos:2 and BUB1B/Phos:3 have opposite effects on the phosphorylation of the kinetochore protein, NDC80 **(**Table 1). Moreover, in an analysis of phosphorylated protein forms that localize to the kinetochore, we found that phosphorylation did not enhance or suppress kinetochore localization *per se*, but did affect the ability of proteins to recruit other proteins to the kinetochore (Table 1). Finally, we found that multiple BUB1B forms form complexes with BUB1 (Figure 5).

### Cross-species comparison of modified protein forms

A related biological question that can be addressed with PRO concerns the cross-species conservation of modified protein forms. Here we described a small scale study involving the phosphorylation of one protein – BUB1B – in three organisms – human, frog, and mouse. Based on the descriptions of BUB1B phosphorylated forms in PRO and a multiple sequence alignment, we concluded that all four BUB1B phosphorylated forms found in humans could be conserved in mice. Three of the four forms are either known to be conserved in frogs or are likely to be, but one form, BUB1B/Phos:4, is not.

Discovery that a modified form found in one species is not conserved in another species is very interesting because a comparison of the function of that protein in the two organisms can provide insight into the role of the modification. Prediction that a modified protein form is conserved in a species where it has not yet been characterized is also useful because it expands the pool of organisms that can be used to study the modified form. For example, confirmation of the existence of BUB1B phosphorylated forms in mice would allow the study of BUB1B forms in mammalian cells undergoing meiosis. These studies could shed light on a question about the function of BUB1B/Phos:1. Frog BUB1B/Phos:1 has been shown to be required for spindle checkpoint cell cycle arrest; in contrast, human BUB1B/Phos:1 is dispensable for cell cycle arrest under these circumstances (Elowe et al., 2007; Wong and Fang, 2007). It is unclear whether this indicates a true difference between the human and frog BUB1B proteins, or if it reflects the fact that the human checkpoint was tested in mitotically growing cells, whereas the frog checkpoint was tested in extracts of oocytes undergoing meiosis. If BUB1B/Phos:1 is indeed present in mice, it would be very interesting to compare its involvement in checkpoint arrest during mitosis and meiosis.

Cross-species analysis of modified protein forms is not limited to a single protein. It can be expanded to include all modified proteins involved in a biological process or present in a particular cellular compartment. It is also not restricted to phosphorylated proteins. The PRO framework can be used to define many kinds of modified protein forms, including those that arise from post-translational modifications such as methylation, acetylation, and ubiquitination and protein isoforms that arise from alternative splicing or from protein cleavage.

### Analysis of evolutionary relationships among proteins

Research into the mechanisms of a biological process often proceeds simultaneously in multiple model systems. In many cases, a clear picture of the process emerges only after data generated from disparate lines of experiment are considered as a whole. Merging of data in this way relies on the assumption that the proteins and pathways examined in the different systems are functionally related. The organization of PRO reflects evolutionary relationships among proteins and can be used as a guide in cross-species comparisons of experimental results. In PRO, organism-specific terms that share 1:1 orthology are grouped under a species-independent parent term (gene-level term) and species-independent terms that share a common domain structure are further grouped under a family level terms. In our analysis, we found that human and yeast BUB1 are 1:1 orthologs and thus share the same species-independent parent terms. However, human BUB1B lies on a separate branch of the PRO hierarchy from its closest yeast relative, MAD3 (Figure 2). Thus, assumptions about the conservation of BUB1 function in humans and yeast are more easily justified than assumptions about the conservation of BUB1B/MAD3 function. Similarly, PRO complexes are grouped under a species-independent complex term if their components are orthologous. Our examination of the BUB1/BUB3 complex revealed that it is conserved in budding yeast, fission yeast, and humans (Figure 5).

### Construction of PPI networks

Often, it is possible to gain insight into the function of proteins in a common pathway by examining their PPIs. PRO facilitates the construction of PPI networks for groups of proteins that are related by some common attribute. Using the built-in PRO search function, it is possible retrieve all PRO terms that share an attribute (e.g., kinetochore localization). The PAF for these terms, which contains PPI information in machine-readable format, can then be downloaded and used to build a PPI network with Cytoscape. Because PRO annotation can show interactions that are dependent on protein modification, PPI networks constructed with PRO have an added dimension that is absent from other PPI network building resources. For example, our PPI network of kinetochore-localized proteins shows that PP2A-B56-alpha interacts specifically with BUB1B/Phos:2 and that PLK1 fails to interact with the unphosphorylated form of BUB1 (Figure 6).

## Conclusion

As we have shown with this use case, PRO is a valuable tool for the study of a complex biological process. Interoperating with other ontologies and resources, PRO provides a structural framework that organizes current knowledge about protein forms, complexes, and cross-species relationships among proteins. While we focused on the spindle checkpoint, the PRO search, display, and analysis strategies we demonstrated here can be applied to any process. PRO-based analysis is particularly valuable for processes where modified protein forms play a prominent role. While PRO coverage is limited for modified forms, we rely on the user community to help in populating the ontology. The web-based RACE-PRO interface provides one means for the user to contribute to PRO. As PRO grows, it will become an increasingly useful resource that can provide insight into biological processes and stimulate the generation of experimentally testable hypotheses.

## Conflict of Interest Statement

The authors declare that the research was conducted in the absence of any commercial or financial relationships that could be construed as a potential conflict of interest.

## Footnotes

^1^http://www.obofoundry.org/

^2^http://www.proconsortium.org/

^3^http://precedings.nature.com/documents/1941/version/1/files/npre20081941-1.pdf

^4^http://www.obofoundry.org/cgi-bin/detail.cgi?id=ncbi_taxonomy

^5^ftp://ftp.pir.georgetown.edu/databases/ontology/pro_obo/

^6^http://mint.bio.uniroma2.it/mint/

^7^http://www.ebi.ac.uk/intact/

^8^http://www.thebiogrid.org

^9^http://pir.georgetown.edu/cgi-bin/pro/race_pro

^10^http://pfam.sanger.ac.uk/

^11^http://sourceforge.net/tracker/?group_id=266825&atid=1135711
